# Editorial: Developing therapeutics for antimicrobial resistant pathogens

**DOI:** 10.3389/fcimb.2022.1083501

**Published:** 2022-11-24

**Authors:** Raja Veerapandian, Parveez Ahamed Abdul Azees, Thiruselvam Viswanathan, Bennett Tochukwu Amaechi, Govindsamy Vediyappan

**Affiliations:** ^1^ Center of Emphasis in Infectious Diseases, Department of Molecular and Translational Medicine, Paul L. Foster School of Medicine, Texas Tech University Health Sciences Center, El Paso, TX, United States; ^2^ Department of Comprehensive Dentistry, School of Dentistry, University of Texas Health Science Center at San Antonio, San Antonio, TX, United States; ^3^ Department of Cellular Biology and Pharmacology, Herbert Wertheim College of Medicine, Florida International University, Miami, FL, United States; ^4^ Division of Biology, Kansas State University, Manhattan, KS, United States

**Keywords:** drugs, anti-virulence drugs, anti-biofilm drugs, repurposed drugs, antibiotic- antibody combination therapy, nanotechnology, *in silico* drug design

The World Health Organization (WHO) declared antimicrobial resistance (AMR) as one of the top 10 global public health issues with an alarming note that the world is running out of antibiotics (WHO, 2017). It is estimated that by 2050 the total healthcare cost for AMR may increase up to US$100 trillion and cause up to10 million deaths each year (de Kraker et al., 2016). A recent study provided the most comprehensive analysis of the burden of AMR to date and highlights that its magnitude on human health is potentially much larger than the major human diseases (Murray et al., 2022). AMR is already a multi-dimensional complex challenge, and the influence of COVID-19 pandemic even worsen the situation. Thus, there is need for safe and effective drugs against AMR pathogens. Given the complexity of AMR and its impact on humans, animals, and the environment, we need multiple approaches to overcome these obstacles (**Figure 1**). As a step towards finding solutions for AMR threat, our Research Topic brings a collection of 11 manuscripts with 8 research and 3 review articles exploring different agents against AMR pathogens.

**Figure 1:**
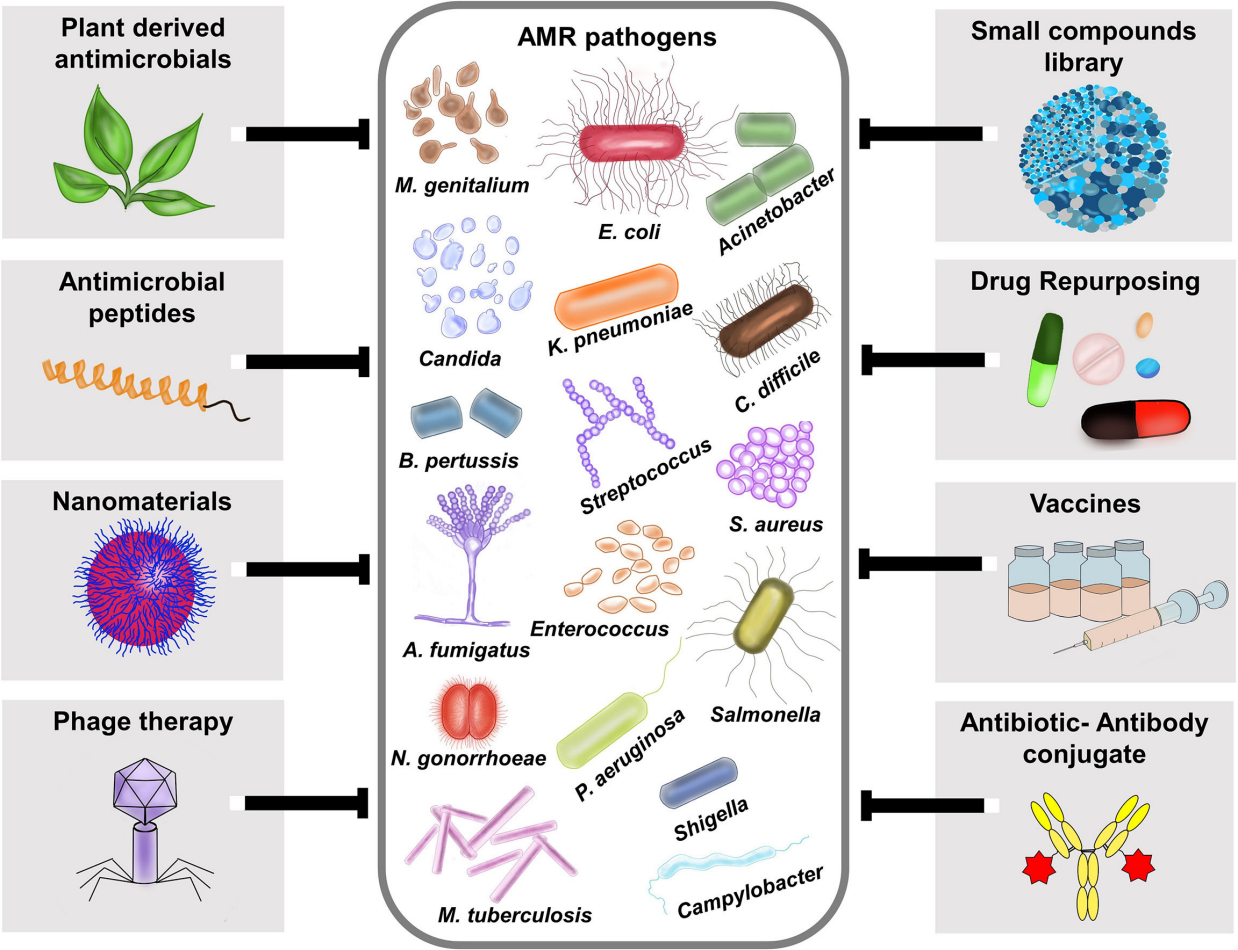
Different therapeutic strategies for combating antimicrobial resistant pathogens. AMR pathogens depicted in the figure is based on the Centers for Disease Control and Prevention (CDC), 2019 antibiotic resistance threats report, which categorized the AMR pathogens into urgent threats, serious threats, concerning threats and watch list https://www.cdc.gov/drugresistance/biggest-threats.html. Various therapeutic approaches like plant- derived antimicrobials, antimicrobial peptides (AMPs), Nanomaterials, Phage therapy, small compound library screening, Drug repurposing, Vaccines, and Antibiotic-Antibody conjugates (AAC) are currently available to fight against AMR pathogens.

Cruz-Lopez et al. have reviewed the efficacy of recently approved antibiotics, β-lactam inhibitors, especially those in advanced clinical phases of development, for treating multi-drug resistant (MDR) infections caused by Gram-negative bacteria. The authors also convey the message that these new drugs should be used wisely. Liao et al., in their review discussed the recent advances in antivirulence drug strategy against *Pseudomonas aeruginosa*, an opportunistic nosocomial MDR pathogen, and elaborates the uses of various drug candidates like small molecules derived from chemical libraries, repurposed drugs, and natural compounds, supplemented by several engineered antibodies, and alginate oligomers against specific virulence mechanisms (*e.g.* T3SS, QS, LPS, etc.).

Plants are the major sources of bioactive products used medicinally since ancient times against infectious diseases (Holzmeyer et al., 2020; Veerapandian et al., 2020). More than 200,000 natural products have been discovered from plants while there are abundant bioactive products yet to be discovered (Hartmann, 2007). Suganya et al. presented a broad and noteworthy review article addressing the use of conventional and complex phytochemicals against MDR bacteria. They have listed the potential bioactive phytochemicals that are effective against MDR pathogens.

Diseases caused by fungi in humans are difficult to manage where mostly they affect individuals with serious underlying illness and compromised host defense (Köhler et al., 2014). Fungi respond to antifungals immediately and treatment failure is common due to antifungal resistance development (Fisher et al., 2018; Fisher et al., 2022). Since fungi are eukaryotes, it is challenging to develop effective antifungals without host cell toxicity. Jothi et al. reported bacterial quorum-sensing molecule (QSM) as promising natural inhibitors against *Candida albicans* virulence, after virtual screening of bacterial QSMs against modeled fungal targets such as Cyr1 and Ras1, which are involved in hyphal growth regulatory pathways. They have validated the hyphal inhibitory effects of the identified compounds through *in silico* analysis and *in vitro* experiments. Recently, many studies have targeted medicinal plants in finding effective drugs against dual species biofilms (Veerapandian and Vediyappan, 2019). A study by Qian et al. has reported the antifungal and antibiofilm efficacy of paeonol, a major phenolic component of Moutan cortex Radicis, the root cortex of *Paeonia suffruticosa* Andrews. Interestingly, this compound has strong antifungal and antibiofilm activities against *C. albicans* and *Cryptococcus neoformans* both as mono- or dual-species. Furthermore, RNA-Seq analysis revealed that the *C. albicans* biofilm was inhibited by downregulating the expression of genes involved in filamentation, adhesion, and growth-related genes. Whereas *C. neoformans* biofilm was inhibited by upregulation of ergosterol biosynthesis-related genes.

An interesting research article from Ramalingam et al. has identified a potential compound from marine actinomycete *Nocardiopsis exhalans*, isolated from the mucus of Scleractinia coral *Acropora formosa*. The purified bioactive fraction of cell-free culture of *N. exhalans* was identified as N-(2-hydroxyphenyl)-2-phenazinamine (NHP), which showed potent biofilm inhibitory activity against *Escherichia coli, P. aeruginosa*, and *Staphylococcus aureus*. Combination antibiotic therapy is found to be superior and may directly or indirectly block the development of antibiotic resistance (Vipin et al., 2020). In this direction, She et al. reported the SPR741, a polymyxin B derivative that has a strong antimicrobial activity against extensively drug-resistant and pandrug-resistant *Klebsiella pneumoniae* when used in combination with macrolide antibiotics (erythromycin and clarithromycin). Yu et al. conducted a meta-analysis of clinical trials comparing novel carbapenem–β-lactamase inhibitor combinations with comparators to assess the clinical and microbiological responses, mortality, and adverse events (AEs). They concluded that imipenem–cilastatin/relebactam (ICR) and meropenem–vaborbactam (MEV) provided better outcome and could be applied as antimicrobial armamentarium against complicated infections. Song and Han have evaluated the Pharmacokinetic/pharmacodynamic exposure of vancomycin, at three infusion modes, intermittent infusion, continuous infusion, and optimal two-step infusion, for MRSA infections in critically ill patients, and reported the optimal two-step infusion mode to be most effective among them.

Antimicrobial peptides (AMPs) are one of the promising alternative solutions for pathogenic microbes that are difficult to treat with conventional antibiotics. AMPs are made up of short amino acid residues, which are present in all species of life (Parthasarathy et al., 2021). To date, more than 3000 AMPs have been reported, out of which >70% show antimicrobial activities (Wang et al., 2016). Wang et al. have reported a potential truncated peptide, named Spgillcin_177–189_ exerting antibacterial activity against several MDR bacterial pathogens. The authors have derived this peptide from the mud crab *Scylla paramamosain*. They showed that the peptide could affect the membrane permeability and cause the accumulation of intracellular reactive oxygen species in the pathogens. In addition, it has strong anti-biofilm activity against *S. aureus* and *P. aeruginosa*. Finally, an interesting study by Chang et al. reported gurmarin, a medicinal plant-derived cyclic peptide that blocks *S. aureus* biofilm formation *in vitro* and *in vivo* (a rat-implant biofilm model). This can be further developed for therapeutic uses. Using biochemical and electron microscopic methods, the authors demonstrated that exposure of the peptide to *S. aureus* diminishes the synthesis of N-acetylglucosamine polysaccharide during biofilm growth. The transcriptomic analysis further shows the upregulation of genes involved in oxidoreductases and the downregulation of genes involved in transferases and hydrolases. The cyclic peptides are known to have diverse bioactivity and are resistant to host proteolytic activity.

Overall, we believe that the articles presented in this collection contribute new knowledge on the discovery and development of antimicrobial agents and constitute a step toward finding potential therapeutics against AMR pathogens.

## Author contributions

All authors listed here made substantial, direct, and intellectual contribution to managing the Research Topic, writing this editorial and approved it for publication.

## Funding

The K-INBRE postdoctoral support to RV and the Innovative Research Award from Johnson Cancer Research Center, KSU to GV are kindly acknowledged.

## Acknowledgments

The authors would like to thank Mahalakshmi Vijayaraghavan, Texas Tech University Health Sciences Center El Paso and Shruthi Vijayaraghavan for their technical support in generating **Figure 1**. We also thank all the authors of the articles and peer reviewers who contributed to this research article collection.

## Conflict of interest

The authors declare that the research was conducted in the absence of any commercial or financial relationships that could be construed as a potential conflict of interest.

## Publisher’s note

All claims expressed in this article are solely those of the authors and do not necessarily represent those of their affiliated organizations, or those of the publisher, the editors and the reviewers. Any product that may be evaluated in this article, or claim that may be made by its manufacturer, is not guaranteed or endorsed by the publisher.
